# Change in basic motor abilities, quality of movement and everyday activities following intensive, goal-directed, activity-focused physiotherapy in a group setting for children with cerebral palsy

**DOI:** 10.1186/1471-2431-10-26

**Published:** 2010-04-27

**Authors:** Anne Brit Sorsdahl, Rolf Moe-Nilssen, Helga K Kaale, Jannike Rieber, Liv Inger Strand

**Affiliations:** 1 Department of Public Health and Primary Health Care, Section for Physiotherapy Science, University of Bergen, PO Box 7804, N-5020 Bergen, Norway; 2 Department of Physiotherapy, Bergen University College, Bergen, Norway; 3 Barnas Fysioterapisenter, Bergen, Norway

## Abstract

**Background:**

The effects of intensive training for children with cerebral palsy (CP) remain uncertain. The aim of the study was to investigate the impact on motor function, quality of movements and everyday activities of three hours of goal-directed activity-focused physiotherapy in a group setting, five days a week for a period of three weeks.

**Methods:**

A repeated measures design was applied with three baseline and two follow up assessments; immediately and three weeks after intervention. Twenty-two children with hemiplegia (n = 7), diplegia (n = 11), quadriplegia (n = 2) and ataxia (n = 2) participated, age ranging 3-9 y. All levels of Gross Motor Function Classification System (GMFCS) and Manual Ability Classification System (MACS) were represented. Parents and professionals participated in goal setting and training. ANOVA was used to analyse change over repeated measures.

**Results:**

A main effect of time was shown in the primary outcome measure; Gross Motor Function Measure-66 (GMFM-66), mean change being 4.5 (p < 0.01) from last baseline to last follow up assessment. An interaction between time and GMFCS-levels was found, implying that children classified to GMFCS-levels I-II improved more than children classified to levels III-V. There were no main or interaction effects of age or anti-spastic medication. Change scores in the Pediatric Evaluation of Disability Inventory (PEDI) ranged 2.0-6.7, p < 0.01 in the Self-care domain of the Functional Skills dimension, and the Self-care and Mobility domains of the Caregiver Assistance dimension. The children's individual goals were on average attained, Mean Goal Attainment Scaling (GAS) T-score being 51.3. Non-significant improved scores on the Gross Motor Performance Measure (GMPM) and the Quality of Upper Extremities Skills Test (QUEST) were demonstrated. Significant improvement in GMPM scores were found in improved items of the GMFM, not in items that maintained the same score.

**Conclusions:**

Basic motor abilities and self-care improved in young children with CP after goal-directed activity-focused physiotherapy with involvement of their local environment, and their need for caregiver assistance in self-care and mobility decreased. The individualized training within a group context during a limited period of time was feasible and well-tolerated. The coherence between acquisition of basic motor abilities and quality of movement should be further examined.

## Background

Optimising participation is seen as the main goal of interventions for children with cerebral palsy (CP). Due to damage of the immature brain, children with CP have disorders of movement and posture development, often accompanied by disturbances of sensation, perception, cognition, communication, behaviour, and by epilepsy and secondary musculoskeletal problems [1]. According to the children and youth version of the WHO's classification (ICF-CY) [2], function can be classified, measured and influenced in several dimensions; like body structure and function, and in activity and participation. In addition, environmental factors as well as the child's health and personal factors may influence the functioning. The relationship between all dimensions are not fully understood [3-7], and which aspects that should be addressed in physiotherapy to promote participation, is an issue of debate.

Whether an intensified training program would accelerate motor development and improve the children's function more than one hour of weekly physiotherapy training as often afforded in Norway is questioned by parents and professionals. Improvement in gross motor function has been indicated after periods of intensive physiotherapy for non-ambulatory children [8], and in children who have practiced functional tasks intensively in their everyday environments [9]. No difference in change of gross motor function has been demonstrated, neither between different intensive approaches [10] nor between training offered in intensive periods versus spread over time [11,12]. Intensive physical training for children has been defined in several ways in recent studies e.g. five sessions a week over six months [11], five sessions a week over four weeks [12], or several daily sessions over five months [9]. There is no consensus regarding the optimal dose of training, and there are only a few studies examining the outcome of intensive physiotherapy training in a group setting [10,13,14].

In physiotherapy approaches like neurodevelopmental therapy (NDT), quality of movement has traditionally been considered important [15,16]. As a reaction to the earlier major focus on quality of movement, a functional task-oriented treatment approach has evolved and is now the preferred therapy [17,18]. However, other interventions like pharmacological, orthoses or surgery, often aim to improve aspects of movement quality [19-21]. In clinical practice it is often presumed that improvements in quality of movement are developed secondary to improvements in basic motor abilities. The importance of quality in movement development has, however, scarcely been investigated although it has been suggested to prevent secondary impairments, decrease effort and increase safety in physical performance.

Valvano [22] has recently described an intervention approach including functional training as well as prevention of secondary impairments by focusing on both activity goals and movement goals. This approach seems to unite several aspects that seem important in therapy for children with CP, and physiotherapy based on this approach should be further investigated.

The aim of the present study was to 1) investigate the impact on motor function and everyday activities of a 3 week-period of intensive, goal-directed, activity-focused physiotherapy in a group setting for children with CP, and 2) examine the relationship between acquisition of basic motor abilities and quality of movements.

## Methods

### Design

A repeated measures design was used in a cohort of children with CP, with three baseline assessments prior to the intervention period and two follow up assessments in the first and third weeks after the intervention (Table 1). The baseline phase, the intervention phase and the follow up phase all lasted for 3 weeks.

**Table 1:** Measures used at the approximate test times.

	Baseline 3 weeks			Intervention 3 weeks	Follow up 3 weeks	
			
*Measure*	Pre-Test1	Pre-Test2	Pre-Test3		Post-Test1	Post-Test2
GMFM	x	x	x		x	x
GMPM		x	x		x	x
QUEST		x	x		x	x
PEDI		x				x
GAS	Goals identified				Goals scored	

GMFM: Gross Motor Function Measure, GMPM: Gross Motor Performance Measure,

QUEST: Quality of Upper Extremity Skills Test, PEDI: Pediatric Evaluation of Disability Inventory,

GAS: Goal Attainment Scaling

### Participants

The total sample included groups of children with CP from four habilitation units in Western Norway who were invited to participate in intensive physiotherapy training for the first time. The children were recruited by the habilitation units, either via advertising in the local newspaper, sending enquiries to physical therapists in community practice or sending invitation to children registered in the unit's files. The inclusion criteria were children with CP in preschool or first years of primary school living within one hour travelling time from the training location. Exclusion criteria were children with other diagnosis than CP and children who, according to the view of professionals at the habilitation units, had extensive strain due to for example repeated hospitalisations or serious health problems in the past year. The habilitation units selected the children and tried to compose groups comprising children with similar functional levels and age, but due to the relatively small number of children with CP in the sites, this was not accomplished in all groups.

The study was performed according to the Helsinki Declaration and approved by the Regional Ethical Committee and the National Data Inspectorate of Norway. Informed consent was obtained from the children's parents before participation in the study.

### Intervention

The intervention model aimed to provide an intensive, but limited period of physiotherapy within the frame of the children's local environment. To ensure that the intervention could fit within the children's everyday life, a model of three hours of training, five days a week in a three-week period was chosen. The training was accomplished in a group setting and based on the following principles: 1) Functional goal directed training [18,22], implying a focus on practicing specific activities of importance to the child, 2) Family centred practice [23], implying that parents were involved in the goal setting process and were active participants in the training, 3) Cooperation between group leaders and parents as well as local professionals and other persons important to the child, to secure carry over of knowledge and skills to the child's everyday life, 4) Applying recent knowledge of motor learning/teaching, e.g. motivating activities, stimulating environments and variation [22,24]. During the baseline and the follow up phases the children continued their ordinary physiotherapy program, mostly one therapy session a week with general goals and emphasis on supervision of the childrens' parents and assistants.

The intervention was developed in collaboration between two researchers and one clinician, and implemented at a private physiotherapy institute for several years before the start of this study. A pilot study with six children was carried out, and based on the result a power calculation was performed [25]. With an alpha of 0.05, a power of 90% and a mean GMFM-66 change score of 2.5 (SD 2.9), the sample size estimate was 14 children. To account for possible drop outs, the sample size was set to minimum 20 children.

Twelve paediatric physiotherapists affiliated to the habilitation units, most with long clinical experience (mean 13 y, range 1-27 y) conducted the group interventions, two for each group with one as a reserve. To make sure that the intervention was conducted similarly, the group leaders participated in a workshop with practical and theoretical lessons, lasting two days. In addition the group leaders were supervised three times during the course of the intensive training, and indication was provided that the group training in the sites were conducted according to the principles of the intervention model.

For a more specific description of the intervention, one group training session in each site was video recorded and the first author (ABS) classified the amount and type of training using a modification of the "Motor Teaching Strategies Coding Instrument" [26]. Based on these recordings the contents of the intervention are further outlined in Additional file 1.

### Measures

Standardized measures found to be reliable and valid, and an individualized measure, were used. In addition there was a qualitative arm of the study, where parents were interviewed. The results are reported in a Norwegian publication [27].

### Primary Outcome Measure

The Gross Motor Function Measure (GMFM-66) [28] was the main outcome measure, and was used to evaluate change in basic motor abilities. The measure reflects aspects of the activity dimension of the ICF-CY. Several studies have documented very good reliability, validity and responsiveness of the measure [29-33]. The GMFM contains 66 test items each scored on a 0-3 ordinal scale. Using the Gross Motor Ability Estimator software [28] a total score (0-100) with interval-level properties is calculated.

### Secondary Outcome Measures

The Gross Motor Performance Measure (GMPM) [34] and the Quality of Upper Extremity Skills Test (QUEST) [35] evaluate quality of gross and fine motor movements, respectively. The measures reflect aspects of the body function dimension and the activity dimension of the ICF-CY. The measures have demonstrated good reliability and validity [36-40]. In the GMPM twenty items derived from the GMFM are assessed on the attributes Alignment, Coordination, Stability, Dissociated movements and Weight shift using an ordinal scale ranging 1-5. Percent scores for the attributes and a total score (scale 0-100) are calculated. The QUEST comprises four domains; Dissociated movements, Grasp, Weight bearing and Protective extension, and 174 sub-items are scored "able to complete ", "not able to complete" or "not tested". Percent scores for the domains and a total score (scale 0-100) are calculated.

The Pediatric Evaluation of Disability Inventory (PEDI) [41] measures functional capacity and performance of children in three domains; Self-care, Mobility and Social Function, and reflects aspects of the activity and participation dimension of the ICF-CY. The PEDI has evidence of very good reliability, validity and responsiveness [31,32,42]. The items of the measure are scored pass/fail and the raw scores of each domain are converted into a norm score or a scaled score. PEDI contains three dimensions; Functional Skills, Caregiver Assistance and Modifications. In this study the scaled scores in the Functional Skills and Caregiver Assistance dimensions were used (scale 0-100; in the Caregiver Assistance dimension higher scores indicate less assistance). PEDI has been translated into Norwegian [42] and is administered in a parent interview.

Goal Attainment Scaling (GAS) [43] was used to capture individualized goal attainment within a predetermined timeframe. For each goal identified by family/child and professionals in collaboration, a scale containing five levels of outcome descriptions are constructed; expected outcome (0), two levels of less (-2 and -1) and two levels of more (+ 1 and + 2) than expected outcome. The scales are scored in a follow up evaluation. When the scales are weighted equally, raw GAS scores can be transformed to standardized GAS T-scores with a mean of 50 and a standard deviation of 10 using the formula [43]:

where *x*_1 _is the attainment score, *n *is the number of scales, and p is the expected correlation of the scales; p = 0.3 suggested reasonable [43]. A T-score of 50 indicates that on average a child s predetermined goals are attained. Validity of the GAS when used in pediatric populations has been demonstrated [44,45].

### Test procedure

All measures were administered and scored by assessors not involved in the intensive training. The assessors and the test locations were the same across all tests for a single child and the tests were to be performed at the same time of the day. Each assessment lasted for 60 to 90 minutes, the child was escorted by its parent(s) and effort was made to put as little strain on the children as possible, e.g. by accomplishing test items in a frame of play, and take breaks when needed. All assessments were video recorded with a handheld camera; the GMPM and QUEST were recorded according to a standard written procedure.

Four paediatric physical therapists (PT) with long clinical experience and familiarity with the measures classified the children according to the Gross Motor Function Classification System (GMFCS) [46] and Manual Ability Classification System (MACS) [47], and performed the assessment with the GMFM-66, GMPM and QUEST. The GMFM was scored by the therapist who administered the measure. The therapist had no access to previous assessments. To obtain sufficient reliability in scoring, the PT used the GMFM-training CD and three of the PTs discussed the administration and scoring in two sessions, each lasting half a day. Validating of the fourth therapist's scorings, who assessed two children, was performed by watching video recordings of the assessments. The video uptakes of the GMPM and QUEST were edited and scored by two therapists who had 22 hours of training as further outlined in Sorsdahl et al. [37]. The edited video clips were blinded to the sequence of the assessments. Two therapists not involved in the intervention (first author ABS and the PT videotaping the tests) constructed the GAS based on goals set by parents/child and the PT who tested the child in collaboration, as suggested when GAS is used as a research instrument [48]. Performances of the selected goal-activities were videotaped at pre-test 3 and at post-test 2. Experienced PTs or OTs from the four habilitation units administered PEDI in interviews with the parents.

### Data analysis

Scores of the GMFM, GMPM, QUEST and PEDI were calculated according to the procedures given in the manuals. In addition, improved and not improved GMFM items during the study period were identified, and two additional GMPM total scores were calculated at pre-test2 and post-test3 for each child; one total score containing GMPM-attributes from the GMFM items that improved during the study period, and one total score containing GMPM-attributes from GMFM items that remain stable. GAS scores were transformed to T-scores [43]. Statistical analyses were performed in Microsoft^® ^Excel Office 2003 and SPSS 15.0 for Windows. Manual calculations of scores and data entry from the scoring forms were accomplished twice to secure data quality. Normality of scores was examined by inspecting Q-Q plots and Shapiro Wilks test for small samples. Repeated measures one-way ANOVA was used for the analysis of GMFM-scores, with time as a five level within-subjects factor. While the assumption of sphericity was not met, degrees of freedom for the F ratio were adjusted according to the Greenhouse-Gessler epsilon [49]. Post hoc analysis was performed, using pared t-tests between measurements with Bonferroni adjustment of the alpha level. In subsequent mixed factorial analyses of variance, the effects of the between-subjects factors with 2 levels; functional ability (GMFCS I-II/III-V), age (3-6 y/7-9 y) and anti-spastic medication (0/1) were included. Analysis of repeated measures was on an intention-to-treat basis, in which missing data were replaced by the last value carried forward [50]. In the secondary outcome measures the normal distribution of variables could be questioned. Repeated measures were accordingly analysed with Friedman's test. Wilcoxon signed rank test was used to analyse difference between pre- and post-intervention scores in GMPM and PEDI. The level of significance was set at p ≤ 0.05, except in the PEDI where p < 0.01 was chosen to adjust for inter correlation between scales [41].

## Results

The study sample included 25 children with CP from five training groups in different parts of western Norway, including a broad spectre of functional disabilities and ages. One child (girl 6 y, GMFCS level II) dropped out during the intervention period due to long travel distance. Two children were excluded: One participated in less than half of the intervention period due to illness (girl 4 y, GMFCS level III), and one was found to have another neurological condition than CP (boy 4 y).

Demographical data of the 22 remaining children are presented in Table 2. Mean age was 5 y 6 m, ranging 2 y 10 m - 9 y 3 m. All levels of the GMFCS and MACS were represented in the sample. Twenty of the 22 children received one to two physiotherapy treatments per week before the intervention period. In addition, nine of the children attended a session of pool therapy weekly. PT from the habilitation units and parents reported that four of the children received BTX-A injections in the lower limbs in the baseline phase, in week one or week three. One child had used a Baclofen pump for 1 1/2 year.

**Table 2:** Participating children (*n *= 22).

Demographic characteristics	
Age (*y m*), mean (range)	5 *y *6 *m*(2 *y*10 *m *-9 *y*3 *m*)
Sex, male/female	15/7
Type of cerebral palsy	
Hemiplegia	7
Diplegia	11
Quadriplegia	2
Ataxia	2
GMFCS	
Level 1	8
Level II	5
Level III	6
Level IV	2
Level V	1
MACS	
Level 1	8
Level II	7
Level III	5
Level IV	1
Level V	1
Associated impairments	
Vision impairment	17
Hearing impairment	2
Seizures	3
Learning disabilities	17

During the group training parents, special educators, assistants and/or local physiotherapists (two to five per child) escorted the children. The children's adherence was high, a mean of 43 of 45 hours (range 39-45) of training in the 3 weeks intervention period. Of all possible assessments, 96% of GMFM-66, 93% of GMPM, 95% of QUEST and 96% of PEDI assessments were completed. Descriptive statistics for the GMFM-66, GMPM and QUEST are shown in Table 3.

**Table 3:** Change in test scores from baseline to follow up.

Measure	*n*	Baseline					Follow up			
			
		PreTest1	PreTest2	Change PreTest1 to PreTest2	PreTest3	Change PreTest2 to PreTest3	PostTest1	Change PreTest3 to PostTest1	PostTest2	Change PreTest3 to PostTest2
										
		Mean (SD) Min-max	Mean (SD) Min-max	Mean (SD)	Mean (SD) Min-max	Mean (SD)	Mean (SD) Min-max	Mean (SD)	Mean (SD) Min-max	Mean (SD)
GMFM-66^1)^	22	61.4 (17.4) 23.4-89.7	61.1 (17.0) 24.7-89.7	-0.3 (2.8)	61.8 (17.4) 23.4-89.7	0.7 (3.0)	65.6 (18.2) 28.0-92.1	3.8 * (4.0)	66.3 (19.4) 25.3-100.0	4.5 * (4.0)
GMPM(A1)	20		58.0 (13.4) 12.0-72.3		59.2 (12.2) 16.0-72.3	1.2 (3.8)	59.5 (12.3) 15.3-70.9	0.3 (3.7)	59.9 (14.1) 12.0-76.7	0.7 (4.6)
GMPM(A2)	20		58.1 (18.5) 12.0-83.2		57.4 (19.9) 12.0-90.2	-0.7 (5.6)	58.5 (18.6) 12.0-84.1	1.1 (6.8)	58.8 (18.8) 12.0-84.1	1.4 (6.5)
QUEST(A1)	20		63.8 (23.6)8.3- 93.6		65.9 (22.1) 8.3-96.1	2.1 (11.6)	69.8 (21.4) 22.7-96.0	3.9 (11.6)	68.8 (25.0) 0.0-97.1	2.9 (11.5)
QUEST(A2)	20		68.3 (24.9) 4.2-97.8		68.4 (25.5) 4.2-97.1	0.1 (6.1)	71.6 (24.8) 2.8-96.9	3.2 (7.9)	71.7 (24.0) 11.1-96.7	3.3 (6.8)

^1)^Post hoc paired comparisons. Paired t-tests with Bonferroni ajustments of alfa level. * p < 0.01.

GMFM: Gross Motor Function Measure, GMPM: Gross Motor Performance Measure, QUEST: Quality of Upper Extremity Skills Test

A: assessor

### GMFM-66

**Figure 1:**
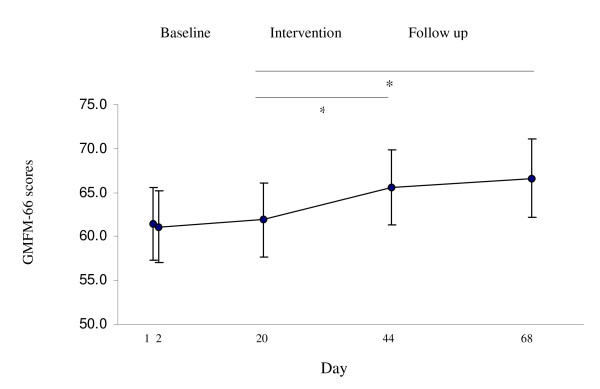
Mean GMFM-66 scores and SEM from baseline and follow up measurements (*n *= 22) * p < 0.01.

A significant main effect for time was demonstrated (F^2^_.7,56.5 _= 17.3, p < 0.01). Post hoc analysis revealed that GMFM-66 scores were rather stable during the baseline phase for the whole sample, but were significantly improved immediately after intensive training (mean 3.8), and was further improved to mean 4.5 after 3 weeks (Figure 1 and Table 3). The main effect of GMFCS-level was significant (F_1,20 _= 28.4, p < 0.01), indicating that children classified to level I-II had higher GMFM-66 scores than children classified to level III-V. There was also a significant interaction between time and GMFCS-level, implying that children classified to GMFCS I-II improved more in GMFM-66 scores over time than children in level III-V (F_4,80 _= 29.7, p < 0.01). There were no significant effects of age or anti-spastic medication.

### GMPM

A total of 1255 items from 20 children were accessible for GMPM video scoring (implying GMFM values > 0). In the editing process 119 items (9.5%) were removed due to poor quality of recording or test administration. Of the remaining GMPM items, the two blinded assessors scored respectively 1132 and 1116. Improved quality of movement during the study period was indicated by positive change scores of both assessors (Table 3), but the improvement was not statistically significant (Chi-square 4.0, p = 0.3; Chi-square 1.9, p = 0.6). There was a significant change in mean GMPM total score for the GMFM items that improved during the study period (GMPM change score 4.7 and 5.9 from first to last assessment for assessor 1 and 2, respectively, p < 0.05), but no significant change in mean GMPM total score for the GMFM items that maintained the same value (GMPM change score 2.1 and 0.9, p = 0.1 and 0.6, respectively).

### QUEST

A total of 11469 sub-items from 20 children were accessible for QUEST scoring, while 928 (8%) were removed during the editing process due to poor quality of recording or test administration. Of the remaining sub-items, the assessors scored respectively 10512 and 10100. Change scores of both assessors regarding quality of upper extremity function were positive over the study period (Table 3), but improvement was not statistically significant (Chi-square 7.2, p = 0.7; Chi-square 6.1, p = 0.1).

### PEDI

Pre- and post-intervention test scores were obtained from 21 of the 22 children's parents, most often from the mothers (81%). The pre-intervention interviews were mainly carried out during the first week of the baseline period, and the post-intervention interviews during the two last follow-up weeks. Significantly improved scores (p < 0.01) were demonstrated in three PEDI domains; Self-care in the Functional Skills dimension and Self-care and Mobility in the Caregiver Assistance dimension (Table 4).

**Table 4:** Test scores of the Pediatric Evaluation of Disability Inventory (scale 0-100) at pre- and posttest (*n *= 21).

	PreTest	PostTest	Change
	
PEDI dimensions and domains	Mean (SD) Min-max	Mean (SD) Min-max	Mean^1)^ (SD)
FUNCTIONAL SKILLS			
Self-care	56.9 (15.9) 11.8 – 81.4	60.9 (18.0) 11.8 – 100.0	4.0 * (4.5)
Mobility	64.7 (21.9) 6.1 – 100.0	67.0 (19.6) 15.2 – 94.2	2.3 (3.8)
Social Function	60.7 (11.6) 31.6 – 89.1	62.7 (13.1) 32.9 – 96.3	2.0 (4.4)
CAREGIVER ASSISTANCE			
Self-care	52.4 (27.0) 0.0 – 100.0	59.2 (26.4) 0.0 – 100.0	6.7 * (10.9)
Mobility	69.1 (25.0) 0.0 – 100.0	72.4 (25.3) 0.0 – 100.0	3.4 * (4.6)
Social Function	68.1 (24.2) 11.3 – 100.0	72.3 (26.9) 0.0 – 100.0	4.2 (7.7)

^1) ^Wilcoxon signed rank test, p-level adjusted for inter correlation in scales, * p < 0.01 (2-tailed)

### GAS

A total of 53 scales with pre-determined goals were developed for the 22 children (one to three goals per child). Thirty-four goals were classified as activity goals, e.g. "Can put trousers on without help ", while 14 were classified as movement goals, e.g. "Can get the heels down when he stands supported", and five were combined. The children's performances at pre-test 3 were set to -2. Three weeks after treatment the goals were obtained in 18 scales (score of 0), and in 17 scales scores of 1 or 2 (more and much more than expected) were achieved. No change was obtained in five scales and less goal attainment than expected in 13 scales. At post-test mean GAS T-score was 51.3 (SD 12.9), implying that on average the children reached the pre-determined goals. Twenty-four (71%) of the activity goals, seven (50%) of the movement goals, and four (80%) of the combined goals were attained.

## Discussion

In the present study change in function was examined in a heterogeneous sample of children with CP who participated in a three-week period of intensive, goal-directed, activity-focused physiotherapy in a group setting. The children's basic motor abilities improved, and predetermined individual goals were the least met in 35 (66%) of the 53 goals. Group training seemed to have a positive impact on the children's and family's daily life since the parents reported that the children's functional skills in self-care at home had increased and their need for caregiver's assistance in self-care and mobility had decreased. The quality of the children's fine and gross motor function, assessed by blinded observers, showed improvement, but not statistically significant. A significantly improved quality of movement, assessed by the GMPM, was found in GMFM items that improved during the study period, but not in items that maintained the same score.

The internationally well acknowledged GMFM was used as the main outcome measure. Change in GMFM-66 scores from the last baseline to the last follow-up assessment after 6 weeks was on average 4.5. Wang et al [51] have proposed a change of 3.71 on the GMFM-66 as a cutoff point between clinical "great improvement" and "not great improvement ". In two studies of intensive physiotherapy where GMFM-66 was used and all GMFCS levels were presented in the samples, the intervention periods lasted for respectively 6 and 30 weeks, with a total amount of 12 and 36 hours of training [12,52]. When comparing results, our outcomes were quite similar or even better, although the intervention period was shorter, but of longer daily duration. In two other studies it was reported that the main change in gross motor function occurred during the first weeks of an intensive training period [9,11]. This could indicate that a higher dose of training should be given over shorter periods of time for achieving increased motor abilities in children with CP. However, there are several factors that could have influenced the results, making comparisons with other studies difficult. The average change in the outcome measures is likely to be dependent on the composition of GMFCS levels and ages in the sample. As seen in our study and also found by others [12,28,53], children classified to level I-II showed the greatest change, as was expected in line with motor development curves for CP [54]. Contrary to the findings of Tsorlakis et al. [53], but in line with Ahl [9], age groups did not have an impact on change in our study. This could be interpreted as the participating children, regardless of age, had not optimised their basic gross motor development before participating in the intensive training. Whether the change in motor abilities in the present study would even out over time as shown in other models of intensive training [11,12], or whether participation in repeated periods of training would result in similar improvement, is a subject of further investigation.

The children's parents and professionals were active participants in the group training. They received much information about the child's motor resources and need for assistance, practical supervision in handling the child and practical ideas to be implemented in the child's everyday environment. Changes in the children's everyday environments and increased skills and knowledge in their caregivers, might have contributed to improved function immediately after the intensive period, and might be an important factor in the maintenance of the child's function in the follow-up phase. In in-dept interviews with thirteen parents performed in the qualitative arm of the study, all the parents underscored their child's functional improvements during and after the intensive training period, and exemplified change observed in the home environments within all dimensions of the ICF, including the environmental and personal factor dimensions [27]. They also reported improved knowledge regarding their child's motor and social functioning, and appreciated to participate in a group setting with the ability to share experiences with other families. From PEDI interviews with parents, we learned that some were uncertain at pre-test about their children's capability of performing tasks, as has also been reported in another study [42]. At post-test, however, these parents seemed much more precise in reporting the children's function in the home environment. The PEDI change scores could thus be a result of change in the children's function as well as increased knowledge and observational skills in the parents.

The individual goal setting for the children was accomplished as a process during the baseline period between parents, child and professionals. In this period the child was repeatedly tested with the GMFM, GMPM and QUEST, and the parents accomplished a PEDI interview, possibly influencing the choice of goals. However, the contents of the goals changed minimally from the first to the last assessment in the baseline period, although the precision increased, implying that parents/child and professionals very early in the baseline period agreed upon goals for the training. Goals explicitly related to e.g. play and social function were less common even if the attainment of many of the goals might contribute in these areas. The potential relationship between movements, activities and participation could possibly be better elucidated to the parents.

The concept of 'Quality of movement' is an issue of debate [15,55-57], and the role of quality of movements in the acquisition of basic motor abilities, has scarcely been elucidated. Improvement in acquisition of basic motor abilities as measured by the GMFM was expected to develop before improvement in quality of movements as measured by the GMPM. However, contrary to our expectations, increase in abilities and improvement in movement quality seemed to occur concurrently during the study period. In basic motor abilities that remained stable, noticeable less change in quality of movement was found. This may be interpreted as improvement in attributes of the GMPM e.g. improved stability or weight shift is a prerequisite for achievement of new basic motor abilities. The finding that assessors tend to judge quality of movement to be improved when abilities of basic motor function improve could also be interpreted as if the assessors confuse ability with quality. One may e.g. be more prone to give higher quality scores when a child rolls all the way from supine to prone, contrary to if the child can only initiate rolling. The difference between the sibling instruments GMFM and GMPM may, in addition, not be straightforward since GMFM clearly captures change in some aspects of quality of movement like weight shift (item 12/13) and stability (item 56). The association between the measures, and acquisition of basic motor abilities versus change in quality of movements, should therefore be further examined.

The video uptakes of the training sessions showed that the intervention was mostly organized as play activities with all the children participating in the same activity, whereas the individualization of training was high. Individual adjustments of the environment and the tasks, along with supervision of the children's escorts, were continuously carried out. When the task motivated the child and the demands on the child was individually tailored, the intensity of the training was high, and the child performed many focused and repeated efforts to complete the task. The experience of being part of a group and of mastering new skills seemed important for the children's motivation, and the group training seemed to give an opportunity for repetition and intensity of training essential for motor learning. It is also interesting to notice that different teaching strategies seemed to be applied when the training was organized as a group activity as opposed to individual activities within the group frame (Additional file 1). Contribution from experienced professionals seemed necessary in order to select appropriate goals, e.g. in line with what a child was just about to manage. Experience was also needed to implement the children's individual goals into a group program and to facilitate motor learning for the individual child during the group session, and at the same time facilitate the group process and supervise the escorts.

Most of the group activities were gross motor activities, but hand motor activities were also included. The QUEST showed improvement after the intervention, but not significant. More specific hand motor training, such as Constrained Induced Movement Therapy has shown significant improvements [58], and may be included in the training or offered in separate periods to give children with unilateral CP more focused hand training.

The repeated measures design of the present study was chosen to take anticipated variability in the children's functioning into consideration, along with considerations regarding feasibility since the intervention was accomplished in five different groups near the children's homes. While the same child is tested in each condition, variability among the children can be measured and separated from error, and smaller but consistent change can be detected as opposed to group designs, where the variability among subjects are uncontrolled and are treated as error. The design is thus advantageous in heterogeneous samples [49,59]. The baseline, intervention and follow up phases were of equal length, giving an opportunity to control maturation effects. Three repetitive assessments were performed at baseline, and showed a rather stable level in the children's gross motor abilities, implying minimal learning effect. This strengthens validity of the change scores in the study. As the GMFM-66 credits new skills [28], improved scores after the intervention, as shown in all children, strengthens the clinical significance of the results. Whether other types of interventions, like practicing tasks in a child's natural environments, are more or less effective than the present intensive group training remains to be shown.

Some parents demand intensive therapy programs for their children, and preferably in a group setting. The children in this study were not randomly assigned, but could be seen as representative of the families who wanted a program of intensive training, the children representing four different geographic areas in Western Norway. The training was well tolerated, but also demanding for both child and family. The children's caregivers appreciated the effort and intensive focus on attainable goals in a restricted time frame. They experienced that the group training was fun and motivating for the children and appreciated that the group was arranged in their local environment [27]. A challenge is to secure that such intensive periods with focused training, are integrated in a child's total habilitation plan.

Little emphasis has been posed on synthesizing evidence regarding physiotherapy for children with CP, in respect to age groups and functional levels, mainly due to small numbers of children and the use of different outcome measures in the studies. What is the optimal dose, content and organisation of the motor intervention in relation to age and functional level are important questions to ask. A model containing two periods of intensive physiotherapy a year for children in pre and first years of primary school, with less focus on motor training in the periods in between, is presently being investigated. Further prospective studies over an extended period of time with careful registration of the children's age, functional level and habilitation services would give accumulated knowledge of the outcomes of different approaches regarding the child and the family in a long time perspective.

## Conclusions

Intensive training in groups for children with CP aged 3 to 9 years resulted in improved basic motor abilities, improved self-care in home environments, and reduced need for caregivers assistance in self-care and mobility. Quality of movement was not changed significantly, but seems to be related to improvement in basic motor abilities. Intensive training focusing on selected, individual activities in a group setting in the children's local environment over a restricted time frame, may seem to be a cost effective and motivating way of optimising function in young children with CP.

## Competing interests

The authors declare that they have no competing interests.

## Authors' contributions

ABS conceived the idea and participated in designing the study, collected and analysed the data and wrote the manuscript. RMN participated in designing the study, analysing the data and drafting the manuscript. HKK and JR participated in developing the intervention, in data collection and were consultants during the study. LIS participated in designing the study, analysing the data, and writing the manuscript. All authors have critically revised the manuscript and approved the final version.

## Pre-publication history

The pre-publication history for this paper can be accessed here:

http://www.biomedcentral.com/1471-2431/10/26/prepub

## Supplementary Material

Additional file 1 **Appendix**. Description of a group session. Click here for file
