# Supplementary material for: Change in basic motor abilities, quality of movement and everyday activities following intensive, goal-directed, activity-focused physiotherapy in a group setting for children with cerebral palsy
Source: BMC Pediatr. 2010 Apr 27;10:26. doi: 10.1186/1471-2431-10-26 (PMC2878295; doi:10.1186/1471-2431-10-26)
Supplement: Additional file 1 — Appendix. Description of a group session. [file 1471-2431-10-26-S1.PDF]

Appendix - Description of a group session

A group session has been described in line with a simplification of the Motor Teaching Strategies Coding Instrument (MTSCI-1) by Larin (2007). The content was described from video recording of one group session, considered representative of the group training. However, depending on the children's goals, ages, functional levels, interests and current state, there are some variations between the groups and day-to-day variations within the groups regarding content, duration of the activities and applied learning/teaching strategies. The amount of training is approximate estimates, and includes small breaks. Transitions between and preparation for activities including undressing/dressing, and one major break with self initiated activities, supplement the described activities.

In the present group training 6 children participated; aged 3 to 8 y; diagnosed with hemiplegia, diplegia or tetraplegia; GMFCS-level I-IV; each accompanied with one escort (parent, assistant or kindergarten teacher). The escorts actively participated in the training e.g. in clapping games, singing, shaking the parachute, ball games. They gave physical and verbal guidance, and took responsibility for security e.g. when children were climbing. The children's goals were related to transitions (e.g. from chair to toilet, from the floor to standing without help, climbing into the sofa at home), maintenance of positions (e.g. sitting, standing, standing on one foot), and different skills (e.g. hopping on one foot, ball-bouncing and ability to climb to the top of the wall bars). In addition some children had goals related to dressing and to hand activities (e.g. more use of affected hand while walking with a walker, carrying a large toy or writing).

Two PTs conducted the group training; one instructed, while the other supervised the children/escorts. The training was conducted in a large room furnished for paediatric activities. Equipment used; exercise mats, stools, wall bars, climbing wall, balance beam, parallel bars, parachute, large boat-swing, prone crawling board, balls, foams of different sizes and shapes, balance cushions, cardstock with song cards, cones, sticks, small toys.

Activity	Task/movement	Reps/Time	Motor Learning and teaching strategies identified during the part (always, often, sometimes, occasionally)
PART 1: GROUP ACTIVITIES ORGANIZED IN A RING			
Welcome songs	Squat-position: Performing arm movements along with the songs	5 min	During part 1:<br>- Visual demonstration (always before and during activities)<br>- Verbal instruction - action (always before and often during activities) e.g. *"Now we are going to be elevators. We push 19th floor and stop, then 38th floor, stop, 101th floor, stop. Ready?" or "Now we are going to stand up. How do we do?"*<br>- Verbal instructions - movements (occasionally during activities) e.g. *"Can you sit with the feet a bit more together?"*<br>- Physical assistance/tactile guidance to children who lacks ability to perform the action (often three children, amount of guidance vary between and within children; occasionally three children)<br>- Children active in creating the activities e.g. choosing songs, deciding which floor the elevator stops, singing,
"Elevator"	Rising from squat to stand. Stop and hold the position three times during the movement trajectory	A total of 15 reps, 2-3 in a set with breaks sitting on a stool in between	
Clapping and finger games	Sitting on a stool: Finger games like "Itsy Bitzy Spider". Clapping to rhymes involving bending and rotating the trunk e.g. clapping on opposite shoulder	10 min	
Clapping games and oral motor activities	Prone lying: Clapping games, waving, pressing a toy that makes bubbles, blowing bubbles	12 min	
Safari	High kneeling and half kneeling. Using arms as	10 min	

	binoculars, looking for animals. Reaching for and holding large animal toys, balancing animals on different parts of body. Dorsal flexion in ankles when crocodile eats on toes		counting, deciding which animal they want, suggesting variations (often)<br>- Using songs, rhymes, short stories and counting for motivation (often)<br>- Using toys and equipment for motivation (sometimes)
Parachute	Sitting, lying and high kneeling: Parachute games like hiding, shaking, para-ball, make balloon fly	15 min	- Motivating feedback e.g. "*Well done"* (occasionally after activities)
Hot-dog	Children are wrapped in a mat, "unwrap" themselves by rolling	5 min	
PART 2: INDIVIDUAL ACTIVITIES ORGANIZED AS STATIONS OR OBSTACLE COURSES			
Individual programs of activities	Ball bouncing, throwing and catching<br>Balance on one foot, different surfaces and heights<br>Crawling over foams of different sizes and shapes<br>Climbing steps or stairs sometimes while carrying a toy<br>Sitting to standing<br>Floor to standing<br>Picking up toys from floor<br>Walk on balance beam, between or over obstacles<br>Climb wall-bars or a climbing wall<br>Crawling over a balance beam fasten to a wall bar<br>Standing with/without support, different surfaces<br>Walking in parallel bars<br>Prone crawling board	3 individual sessions during the training, each lasting 10-15 minutes	During part 2:<br>- Verbal instruction from escort (often before and during activities). Action or movement instructions dependent of the escort<br>- Physical assistance/tactile guidance (occasionally or never depending on the activity)<br>- Using toys and equipment for motivation (always)<br>- Motivating feedback e.g. "*Well done" "Come on!"*(often during activities)<br>- Feedback on result *"You are standing!"* (occasionally after activities)<br>- Visual demonstration (occasionally before activities)
PART 3: CLOSING ACTIVITIES			
Stretching	Muscles in legs and arms	8 min	During part 3:<br>- Verbal instruction - movement (always before stretching)<br>- Visual demonstration (always before stretching)
Large boat swing	Climbing into. Sitting or standing in the swing	10 min	- Verbal instruction - action (always –swing)<br>- Songs, music CD and counting for motivation (always)
Reflections/comments	Each child comments on the training	5 min	- Physical assistance (always during stretching, two children when climbing into the swing<br>- Children active in creating the activities e.g. choosing songs, singing, counting, deciding when enough stretch (sometimes during activities)<br>- Motivating feedback e.g. "*Well done*"(occasionally after activities)
